# Deinstitutionalization from the perspective of community-dwelling adults with a severe mental illness in Amsterdam: a cohort study protocol

**DOI:** 10.1186/s12889-022-13291-w

**Published:** 2022-05-12

**Authors:** Menno Segeren, Steve Lauriks, Martijn Kikkert, Jet Heering, Nick Lommerse, Gwen van Husen, Arnoud Verhoeff

**Affiliations:** 1 Public Health Service Amsterdam, Department of Epidemiology, Health Promotion and Care Innovation, Amsterdam, the Netherlands; 2 Department of Research, Arkin Mental Health Care Institute, Amsterdam, the Netherlands; 3 GGZ inGeest, Department of Research & Innovation, Amsterdam, the Netherlands; 4 Department of Sociology, University of Amsterdam, Amsterdam, the Netherlands

**Keywords:** Deinstitutionalization, Severe mental illness, Community-dwelling, Recovery, Quality of life, Societal participation, Cohort study, Study protocol

## Abstract

**Background:**

People with a severe mental illness (SMI) increasingly receive ambulatory forms of care and support. The trend of deinstitutionalization accelerated in the Netherlands from 2008 and onwards without sufficient understanding of its consequences. The study protocol herein focuses on deinstitutionalization from the perspective of adults with an SMI living within the community in Amsterdam and aims at delivering better insight into, amongst others, their recovery, quality of life, societal participation and needs for care and support.

**Methods:**

A cohort design will be used. A representative sample of community-dwelling adults with an SMI, including those in care (*n* = 650) and not in care (*n* = 150), will be followed over time. During a two-year time period, participants will be interviewed twice using a wide-ranging set of validated instruments. Interview data will be matched with administrative data about the care process, as retrieved from their patient files. Primary outcomes are changes over time in recovery, societal participation and quality of life, controlled for the occurrence of adverse life-events during follow-up. Additionally, prevalence estimates of and associations between social functioning, safety and discrimination, substance use and health indicators will be investigated.

**Discussion:**

The study protocol aims at delivering a comprehensive insight into the needs of community-dwelling adults with an SMI based on which ambulatory care and support can best be provided to optimally promote their social recovery and well-being.

## Background

In many high income countries, including the Netherlands, an increasing proportion of people with a severe mental illness (SMI) live independently, within the community. Instead of residing in a long-stay clinical setting in psychiatric institutions, these ‘outpatients’ receive ambulatory mental health care in addition to social and economic support when required. This trend is historically and internationally referred to as deinstitutionalization. In the Netherlands, deinstitutionalization gained new impetus in the year 2008 and onwards. In 2013, the Dutch Ministry of Health, Welfare and Sports, the mental health care sector and health insurance companies agreed to reduce the number of clinical psychiatric beds designated to people with an SMI by a third in the year 2020, with the bed-count in 2008 being the reference [1, 2]. Two underlying rationales guided the agreement. First, ambulatory mental health services at home would be more beneficial with respect to recovery of people with an SMI, specifically social and personal aspects of recovery. Second, ambulatory mental health services at home would be more cost-effective than the provision of long-stay inpatient care services.

In parallel, a number of changes in legislation transferred the responsibility regarding the public mental health care system (Social support act. In Dutch: *Wet maatschappelijke ondersteuning* 2015) [3] and the social inclusion of vulnerable groups (Social support act; Participation act. In Dutch: Participatiewet 2018) [4] from the national government to municipal (local) governments. Municipalities were tasked with two new responsibilities, namely providing ambulatory support to vulnerable populations (e.g., nursing care for home-dwelling elderly, reintegration of the long-term unemployed, and social recovery of persons with mental and/or physical disorders and disabilities) and developing and installing local policy measures required to guide the process of deinstitutionalization.

As such, whereas until fairly recently the treatment of people with an SMI primarily focused on improving their mental and physical functioning, present-day treatment plans dedicate additional focus to building and maintaining a satisfactory informal and formal social life [5, 6]. The extent to which people with an SMI are able to satisfy such social needs depends strongly on the support that relatives/friends and, on occasion, mental health care professionals are willing and able to provide. For varying reasons, however, a considerable proportion of people with an SMI are unable to capitalize the (informal) social support that is offered to them.

A vast literature shows that people with an SMI, especially those living within the community, constitute a vulnerable population with respect to several adverse, and often intertwined, outcomes. Pivotal is that people with an SMI are prone to experience difficulties regarding social participation (i.e. (paid) work and education) [7, 8] and social inclusion [9], which in itself are known to be associated with, amongst others, quality of life and the extent to which their health care and support needs are met [10, 11]. Within the target population of people with an SMI, a substantial proportion of people present with a psychotic or mood/anxiety disorder, while substance use disorders are also highly prevalent (i.e. dual diagnosis disorder) [12], associated with unhealthy life-style behaviors [13], poor physical health and overall suboptimal well-being [14]. Importantly, people with an SMI are also a high risk group with respect to experiencing discrimination [15, 16], stigmatization [17, 18], social exclusion [19] and victimization [20–24], each of which is detrimental to their social participation. Such outcomes are strongly associated with the concept of recovery of people with an SMI, which is increasingly broken down into clinical, functional and personal recovery [25–27]. Clinical recovery concerns the degree of symptomatic symptomatology [28]. Functional recovery refers to functional outcomes with respect to important life-domains such as work, daytime activities, societal participation, housing, social relations and daily living tasks. Personal recovery, or subjective recovery, refers to living a meaningful life such as captured by the CHIME-framework: connectedness, hope and optimism, identity, meaning in life, and empowerment [29]. The vulnerability of this target population constitutes the current responsibility of local government (i.e. municipalities), in collaboration with private care and support services, to provide this group with the necessary support in order to maintain satisfactory living conditions. In this respect, deinstitutionalization of people with SMI concerns more than the mere redesign of mental health care services. Its impact is much wider and multifaceted and it affects a large variety of stakeholders involved in the target population of people with SMI. Deinstitutionalization requires each of these stakeholders to reassess their role, interest and position in local care and support networks for people with an SMI.

Despite evident changes that are expected to result from the ongoing deinstitutionalization in the Netherlands, there is not yet a clear view on its effects and implications from the perspectives of the most important stakeholders. These primary stakeholders are 1) people with an SMI themselves, 2) their relatives and friends (i.e. informal providers of care and support), 3) mental health care providers and social services including their professionals, 4) neighborhoods and fellow residents, and 5) local government. Some recent initiatives and research projects have already been carried out to scrutinize the consequences of deinstitutionalization in the Netherlands. However, they did so primarily from a national or regional perspective [30–33]. Notwithstanding their merit, these studies did not address the specific questions and topics concerning deinstitutionalization that emanate from the combined perspectives of each of the aforementioned stakeholders and from the level of the municipality.

Against this background, the public health service of Amsterdam, in close collaboration with Amsterdam’s two largest mental health care institutions, initiated a research program ‘Deinstitutionalization Amsterdam’ (in Dutch: *Ambulantisering Amsterdam*). This research program aims at providing more insight in the process, results and effects of deinstitutionalization from the perspectives of people with an SMI, their close relatives and friends, their neighbors and neighborhoods, the mental health care institutions, social services and welfare organizations involved, and the municipality. The main question of the research program is: *How can ambulatory care and support for community-dwelling adults with a severe mental illness best be provided to optimally promote their social recovery?*

The research program is modular. It consists of multiple interrelated research projects. Each of these projects addresses deinstitutionalization from one of the aforementioned perspectives. The current study protocol applies to the ‘client study’ of the program, that focuses on the perspective on deinstitutionalization from its main stakeholder: community-dwelling adults with an SMI. More specifically, the protocol lays out the study design by means of which a representative research cohort of people with an SMI in Amsterdam will be assembled (i.e. inclusion and exclusion criteria) and followed over time. Additionally, the protocol describes the process of participant recruitment, the instruments that will be administered, the study’s primary outcome measures and the statistical analyses that will be performed. Furthermore, the protocol specifies how the following research questions will be addressed:

1. Which trends in the development of community-dwelling people with an SMI concerning their quality of life, recovery, daily functioning and societal participation can be observed over the course of time?
2. Which needs of community-dwelling people with SMI are most important with respect to their recovery and societal participation?
3. How are these needs addressed and accommodated by formal and informal providers of ambulatory care and support?
4. How can the provision of community-based ambulatory care for people with an SMI be described with respect to treatment compliance, therapeutic alliance and frequency/timeliness of care consults?
5. What is the prevalence and incidence during follow-up of personal crises and escalations, including psychological crises, compulsory (clinical) treatment and victimization, among this population?

## Methods

### Study design

The Deinstitutionalization Amsterdam Client-study is a cohort study that started in December 2017. The study is planned to include approximately 800 adults with an SMI. The study population is designed to be representative for the target population of community-dwelling adults with an SMI at the city level, the level of the city-districts and the level of the city’s quarters (i.e. combined neighborhoods). To acquire a better insight into developments in the functioning and recovery of community-dwelling adults with an SMI and their associated needs for care/support, a comprehensive set of measurement instruments will be administered twice, with a two-year time interval, by means of semi-structured interviews. Also, the occurrence of adverse life-events during follow-up will be associated with developments in people’s functioning (i.e. social recovery and societal participation) over the course of time. Additionally, information about the care processes during that time will be retrieved from participants’ patient files.

### Setting

The study is situated in Amsterdam, the Netherlands. Internationally, the prevalence of SMI in the general population is estimated to vary between 0.5–2.9% according to, amongst others, population density, age distribution, social economic indicators, the number of (ethnic) minorities and the level of social cohesion [34–38]. Concerning specifically Amsterdam, reliable figures about the number of inhabitants with an SMI are lacking. The most recent 12 month-prevalence estimates vary between 1.6% [31] and 2.0% [30, 39], equaling an SMI population of between 7923 and 9827 adults within the municipality, of whom approximately 90% are considered community-dwelling and 10% to reside in long-term clinical settings [40].

The city of Amsterdam is subdivided into 8 administrative districts of which 7 residential districts and 1 industrial/harbor district. Together, the residential districts consist of 476 distinct neighborhoods, conglomerated into 99 quarters (or combined neighborhoods). Participants will be sampled and recruited at the level of quarters. As such, the resulting sample is designed to be representative at the level of the city, the city-districts and the quarters. From each of the residential districts, the two largest quarters are selected (based on population size, ranging from 12,946 to 26,787 registered residents). Together, these 14 quarters comprise 104 neighborhoods, 4437 unique postal codes and 237,157 inhabitants [38]. Within these quarters, prevalence estimates of SMI range between .02 and 3.08% [36] and an average SMI prevalence of 1.6% of the adult population of these quarters is assumed.

### Participants

Participants will be recruited on the basis of three inclusion criteria: age, SMI and location. The age-criterion is chosen to align the study population with the patient populations of mental health care providers and care system for adults. Although deinstitutionalization also applies to minors (< 18) and the elderly (65+), these populations are serviced by care providers in other care networks: youth [41] services and elderly [41] care, respectively. Integration of the care systems for these different age-groups is, at the time of this writing, still in its infancy. The scope of the current study is therefore limited to adults only (age 18–65).

The SMI criteria as postulated by the ‘Dutch consensus group EPA’ [31] (EPA is the abbreviation of Ernstige Pychiatrische Aandoeningen which translates to SMI) are applied. These criteria are as follows:

- there has to be a psychiatric disorder that requires care/treatment;
- the disorder is directly related to severely impaired social/societal functioning;
- the functioning impairments are both cause and effect of the psychiatric disorder at play;
- the disorder is not transient in nature (i.e., it is structural or chronic for at least 2 years);
- coordinated care from professional care providers, embedded in local health care networks, is indicated to perform and effectuate the treatment plan for the psychiatric disorder.

This SMI-definition matches international SMI-criteria to a large extent [35, 36, 42], but not completely. Although a consensual definition is SMI is lacking, SMI is commonly understood as a function of three “D’s”, diagnosis, disability and duration [43, 44]. In contrast, the definition used in the current study focuses on the functional impairments and its effects on daily activities for which people with SMI need care and support. This results in a more inclusive definition of SMI in which no diagnosis is a priori excluded or included. More specifically, certain psychiatric symptoms, such as those associated with schizophrenia spectrum disorders (e.g., delusions and hallucinations), are considered to belong exclusively to the target population of people with an SMI. Other symptoms have been demonstrated to occur at different rates among people with an SMI. For example, poly drug use, personality disorders and pervasive developmental disorders are prevalent symptoms among people with an SMI as well. Mood and anxiety disorders and alcohol abuse, on the other hand, are relatively infrequent [34]. In addition, people with an intellectual disability or a physical impairment and people with dementia may also meet the aforementioned SMI-criteria. However, they do so only if a normative need for mental health care regarding psychiatric comorbidity is established.

#### Exclusion criteria

The following exclusion criteria will be applied:

- people with an intellectual and/or physical disability who are not in need of mental health care for co-morbid psychiatric disorders;
- people who suffer from dementia and who are not in need of mental health care for co-morbid psychiatric disorders;
- people who meet all inclusion criteria but whose formal postal code of their home address matches those of long-term clinical locations of mental health care providers (i.e. not community-dwelling patients);
- people whose proficiency in either Dutch or English is insufficient to be interviewed, and people who cannot be approached due to other mental health or social problems.

Within the larger population of community-dwelling adults with an SMI, the study differentiates between three cohorts. First, the ‘in-care cohort’ consists of adults with an SMI who receive ambulatory mental health care. Second, the ‘not-in-care cohort’ is formed by adults with an SMI who do not receive specialized mental health care from a formal mental health care provider yet may receive social welfare/support. Third, the ‘LZA-cohort’ are participants of a former longitudinal cross-sectional study into long-term care-dependent psychiatric patients in Amsterdam, carried out in 2005 and 2012 by Amsterdam’s largest mental health care providers Arkin and GGZ inGeest (in Dutch: the Langdurige zorgafhankelijkenstudie or LZA-study). This study focused on the psychological and social functioning and quality of life of people with a chronic SMI [32, 45]. Participants of the LZA-study who provided written consent to be re-approached were eligible to participate in the current study. The reason to include this LZA-cohort is that the current study utilizes a similar set of instruments which enables a third measurement for the LZA-study, providing insight in long-term (2005–2020) developments of this subgroup within the population of people with SMI.

With respect to the in-care and not-in-care cohorts, their relative proportions within the total SMI-population in Amsterdam are approximately 76 and 24%, respectively [30]. Furthermore, around 90% of both groups lives independently (i.e. community dwelling). In-care participants will be recruited at the two largest mental health care providers in Amsterdam, Arkin and GGZ inGeest. Together, these organizations are estimated to provide care/treatment to two thirds of the total in-care population of people with an SMI in Amsterdam [39]. The remaining 33% of the in-care SMI-population, those who receive mental health care from one of several other (small) providers, will not be included as we expect to obtain a representative sample of the in-care population from the selected service providers. Moreover, recruiting participants at numerous care providers would require a disproportional effort and is therefore considered unfeasible. The ‘not-in-care’ participants will be recruited at a variety of social welfare and social support organizations in Amsterdam, including organizations that target the homeless (e.g., HVO-Querido, Leger des Heils, Cordaan, Volksbond). In this group, the SMI-criteria will be assessed during the interview.

**Fig. 1:**
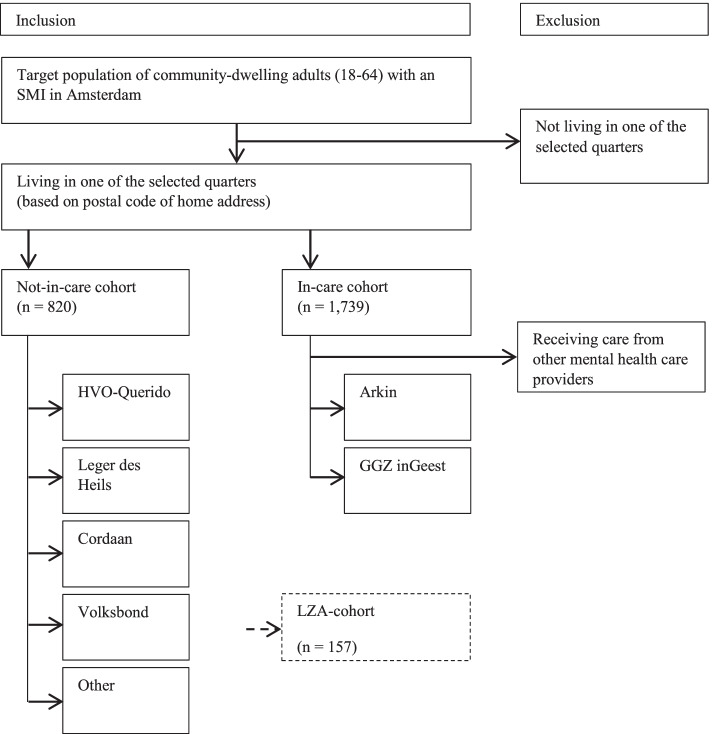
Flowchart of the composition of the target population from which the study population will be sampled

As such, on the basis of 1) the assumed average SMI prevalence of 1.6% among the adult population of the selected quarters (237,157 * 1,6% = 3795 people), and 2) the proportion of adults with an SMI who are registered as a mental health care patient (76%, *n* = 2884), and 3) of whom at one of the two main mental health care providers in the city (66%, *n* = 1903) and 4) the proportion within this group who live independently (i.e. community dwelling) (90%), the in-care cohort is estimated to consist of 1739 adults with an SMI. On the basis of the aforementioned proportions, the not-in-care cohort is estimated to consist of 820 people. The LZA-cohort potentially consists of 157 former LZA-participants who gave permission to be re-approached. Based on expected response rates of 33% in the in-care cohort, 20% in the not-in-care cohort and 50% in the LZA-cohort, the study sample will consist of approximately 800 participants. Figure 1 presents a flowchart depicting the composition of the study population.

In-care and not-in-care participants were recruited consecutively, to avoid duplicate recruitment attempts. The recruitment period of the in-care group, and the LZA-cohort in parallel, was December 2017–January 2021. Recruitment of participants in the not-in-care group was executed between January 2021 and January 2022. At baseline, participants were interviewed about their functioning with respect to important aspects of life (see instruments) by a pool of trained interviewers. All participants will receive a financial compensation of €15, for each interview. Interview data will be matched with information retrieved from patient files that concerns important aspects of the care process. Follow-up interviews will be held two years after the first interview and will consist of the same set of instruments as administered during interview 1. The recruitment period of follow-up interviews of the in-care cohort and the LZA-cohort will start in December 2019 to end in June 2022. Not-in-care participants will be approached to participate in interview 2 between January and September 2023.

### Recruitment

#### Recruitment in-care cohort at Arkin

Within Arkin, several teams are selected from which patients with an SMI will be asked to participate. The teams included are Flexible Assertive Community Treatment teams (FACT), basic mental health care teams (BMHC) with a focus on more stable patients with a chronic mental health problem, forensic FACT-teams of the Forensic Ambulatory Care (FAC) and “Jellinek” Outreaching Teams (JOT) for substance dependency [46]. FACT-teams provide multidisciplinary ambulatory care for the treatment and guidance of people with an SMI. BMHC-teams provide general ambulatory treatment that involves long-term guidance with respect to relapse-prevention, monitoring and care-coordination for stabilized and low-complexity chronic psychiatric patients at low risk of psychiatric crisis. The target population of FAC is primarily a forensic population. FAC-teams offer both voluntary and involuntary treatment. FAC-patients are typically diagnosed with psychiatric, addiction and/or personality disorder(s) and multi-problem situations (that include social and judicial problems). FAC-teams provide ambulatory outreaching care and outpatient treatment. JOT-teams target people with functioning impairments that result from severe addiction problems and (often) comorbid disorders. JOT provides ambulatory multidisciplinary care.

Participants are selected at their location of care. Primary caregivers (i.e. case managers or psychiatrists) will assess and advise about eligibility for participation and a preferred approach-strategy. When direct approach is advised, a research assistant will contact these patients to inform them about the study and to invite them for participation. Caregivers that prefer to first inform their patients themselves will hand out a folder and inform their patient during the next consult. Only patients who wish to participate will be contacted by a research assistant to schedule an interview appointment. Patients who temporarily meet an exclusion criterion will be re-approached at a more suitable time, as advised by their caregiver. Interviews will be conducted at the location of care, an interview cubicle at the Public Health Service Amsterdam or, if possible, at home. Informed consent is given prior to the start of the interview. In case of no-shows without an explicit wish to withdraw from the study, patients will be approached for a new appointment. In case of no-show due to sickness or mental instability, a new appointment will be scheduled several weeks into the future.

#### Recruitment in-care cohort at GGZ inGeest

GGZ inGeest will recruit participants at several of their mental health care departments. These are selected on the basis of their distinct care facilities and specific types of treatments that are commonly provided to people with an SMI. Patients from regular FACT-teams will be selected as potential participants. Additionally, participants will be selected at mental health care outpatient clinics whose patients are either stabilized with respect to medication use and crisis management or who receive specialized care for distinct psychiatric diagnoses (e.g., bipolar disorder).

After the research assistant has given an oral presentation of the study, primary caregivers will be asked to select and recruit patients eligible to participate. Patients who wish to participate can inform their caregiver and will be approached by telephone by a research assistant. Further explanation of participating in the study will be provided by sending an information brochure by mail. Then, participants will be contacted to schedule an interview appointment.

#### Recruitment not-in-care cohort

Not-in-care participants will be recruited at a variety of front-office, social support and primary care services. These include homeless shelter services (*n* = 3), financial and administrative support services (*n* = 1), social care services (*n* = 2), and integral community support teams (*n* = 7). Due to uncertainty about meeting the first three SMI criteria, professionals from these services will be asked to select participants from their caseload based on client histories and their professional assessment with regard to meeting specifically the last SMI criterion of the study (i.e. coordinated care from professional care providers, embedded in local health care networks, is indicated to perform and effectuate the treatment plan for the *assumed* psychiatric disorder). A definitive validation of meeting the inclusion criteria with respect to SMI will be made on the basis of their responses on a selection of items concerning psychiatric symptoms in the questionnaire, current levels of general functioning and mental health treatment history. As such, it is possible that people who participated in interview 1 may still be excluded from the final study sample (they will receive an incentive for their time and effort).

#### Recruitment LZA-cohort

Recruitment of former participants from the LZA-cohort will go in parallel to recruiting the in-care cohort at Arkin and GGZ inGeest, with the exception that the location criterion is not applied to this group. LZA-participants who are still in care at Arkin or GGZ inGeest will be added to the larger in-care cohort and no separate recruitment procedure is formulated. LZA-participants who are no longer in care at Arkin or GGZ inGeest will not be re-approached. LZA-participants who live in a clinical setting for a prolonged period of time will be approached by the fieldwork coordinator of the initial LZA-study. A more detailed description of the recruitment protocol of the LZA-study can be found in earlier publications [32, 45].

### Instruments and variables

#### Interview data

The current study focuses on the functioning of people with an SMI in important life-domains on the basis of five main themes: 1) sociodemographic characteristics, 2) social functioning, 3) safety and discrimination, 4) substance use and life events, 5) subjective appraisal and recovery and 6) health. Each of these themes is addressed by distinct outcomes, that will be assessed with validated instruments. The selection of instruments included matches the instrumentation of the LZA-study to a large extent, thereby enabling a third wave of the LZA study. Table 1 present an overview of all instruments included in the study protocol.

**Table 1:** Overview of the instruments included in the questionnaire

Domains and outcome measures	Instrument	No. items
Social functioning		
Social network	Social network questionnaire (SNQ)	14
Social support	Inventory for social support (ISS)	11
Loneliness	Loneliness scale (De Jong-Gierveld)	6
Social participation	Items from health of the nations outcome scale (HoNOS)	4
Social exclusion	Social exclusion index (SEI)	19
Safety and discrimination		
Subjective safety and victimization	Integral safety monitor (ISM)	27
Discrimination	Daily discrimination scale (DDS)	9
(self)stigmatisation	Stigma scale (S-scale)	28
Substance use and life events		
Substance use and dependency	Measurement for triage and evaluation (MATE)	9
Alcohol use disorder	Alcohol use disorder identification test (AUDIT)	10
Substance use disorder	Drug use disorder identification test (DUDIT)	10
Negative life-events	List of threatening experiences (Brugha)	12
Subjective appraisal and recovery		
Quality of life	Manchester short assessment of quality of life (MANSA)	16
Recovery	Integral recovery scale (symptomatic, social, personal) (IRS)	13
	National recovery scale (personal recovery)	26
Health		
Physical health	RAND SF-36 Health Survey	12
Needs for care/support	Camberwell assessment of need short appraisal schedule (CANSAS)	25
Psychopathology severity	Brief psychiatric rating scale (BPRS)	24

##### Sociodemographic variables

Sociodemographic variables included are age, gender, cultural-ethnic background, education status, living situation (e.g., alone, with a spouse and/or children, in a group, with parents), type of housing, work/education/daytime activities (i.e. societal participation) and family situation.^1^

##### Social functioning: social network

The Social Network Questionnaire (SNQ) [47] will be used to determine the size and composition of one’s social network. The 14-item instrument covers several categories of social contacts (e.g., family, work, education) and counts the total number of social contacts reported and the number of concrete social contacts in the preceding month. The instrument distinguishes between one’s primary and secondary social network. The SNQ has been tested for validity and reliability (alpha = .82) [48] in determining network size and frequency of contacts.

##### Social functioning: social support

The Inventory for Social Support (ISS) is a self-report instrument that measures social support [49]. More specifically, the 12-item instrument measures subjectively experienced support received from one’s most important social contact. The ISS distinguishes between emotional and practical support. Psychometric properties of the ISS are satisfactory, as demonstrated by its internal consistency and convergent and divergent validity [50].

##### Social functioning: loneliness

Feelings of loneliness will be measured with the short version of the loneliness-scale [51, 52]. The instrument measures the extent to which one’s social contacts satisfy one’s need for social contact/interactions. Participants indicate the extent to which they agree with 6 statements (e.g., *I miss having people around me*, *There are plenty of people I can rely on when I have problems*) with a score ranging from 1 = *No!* to 3 = *sort or less* to 5 = *Yes!*. The instrument includes a total loneliness score and two subscales: social loneliness and emotional loneliness. The loneliness scale and its’ subscales have been shown to be reliable and valid in several countries [51] and among several ethnic target populations [53].

##### Social functioning: social exclusion

The Social Exclusion Index for Health Surveys (SEI-HS) is a 19-item instrument [54–56] designed to measure social exclusion according to the following subdomains: social participation (e.g., *I feel isolated from other people*), material deprivation (e.g., *Does your household have enough money to visit family or friends?*), access to basic social rights (e.g., *How satisfied or dissatisfied are you with the quality of your home?*), normative social integration (e.g., *I occasionally do something for my neighbors*) and perceived social exclusion (e.g., *the feeling of being isolated from society*). A person is socially excluded in case of accumulation of disadvantages in four subdomains. The SEI-HS has been demonstrated to have adequate internal consistency for the general index and satisfactory construct validity [57].

##### Safety & discrimination: subjective safety and victimization

Victimization will be measured with the Integral Safety Monitor (ISM) [58]. The instrument is used in the context of a yearly national monitor study of the Dutch Ministry of Safety and Justice, performed by Dutch municipalities. The ISM is a self-report instrument that includes the assessment of neighborhood livability, subjective safety and victimization of 15 different types of crimes among which violent crimes (e.g., battery/assault, sexual violence), nonviolent crimes (e.g., theft, home burglary) and cybercrimes (e.g., identity theft, cyber bullying). The IVM has, to our knowledge, not been tested for its psychometric properties. However, it is considered an adequate and well-structured instrument to gather self-reported information about victimization [58]. Self-report is considered to be more reliable and accurate than using information from objective registries that tend to underreport victimization (e.g., police, public prosecutor) [59].

##### Safety & discrimination: discrimination

The Dutch translation of the scale for daily discrimination of the National Survey of “Midlife Development in the United States” [60, 61] will be used to assess personal experiences with discrimination. The scale includes 7 types of discrimination, that are assessed with 9 Likert-type items (e.g., *How often do you experience being treated less politely than other people)* with answer categories ranging from 1 = *often* to 4 = *never*. Levels of daily discrimination are measured according to a total score of the recoded 9 items with higher scores indicating higher perceived discrimination. The internal consistency of the discrimination scale is high (alpha = .97) [61].

##### Safety & discrimination: (self)stigmatization

The stigma scale [62] is a 28-item self-report instrument for the measurement of (self) stigmatization and perceived discrimination among people with a mental illness. The scale has a three-factor structure including discrimination, disclosure and positive aspects of mental illness. Items (e.g., *I have been discriminated against in education because of my mental health problems, I do not mind people in my neighborhood knowing I have had mental health problems, People have avoided me because of my mental health problems*) are rated on a five-point Likert scale ranging from 0 = *strongly disagree* to 4 = *strongly agree*. Higher scores indicate higher perceived stigma. The S-scale has a test-retest reliability of .71 and good internal consistency (Cronbach’s α = .87) [62]. Content validity and construct validity are sufficient [63].

##### Substance use & life events: substance use

The Dutch version of the Measurement in the Addiction for Triage and Evaluation (MATE) [64] will be used to assess substance use [65]. Substances included are alcohol, nicotine, cannabis, opioids, cocaine, stimulants, ecstasy/MDMA, GHB, other hallucinogens, gambling and sedatives. Items assess the number of days on which these substances have been used in the past 30 days, and amount of use on a typical day of use. The MATE is a structured instrument that is scored on the basis of an interview with the patient. The MATE has been demonstrated to be reliable and valid, however more convincingly for Europeans [64].

In addition to the MATE, the Alcohol Use Disorder Identification Test (AUDIT) [66] and the Drug Use Disorder Identification Test (DUDIT) [67] will be used to assess alcohol and drug dependency, respectively. The AUDIT (10 items) and DUDIT (11 items) are screening instruments that assesses frequency/quantity of intake, hazardous alcohol/drug use and dependency symptoms. Scores on the AUDIT and DUDIT range from 0 to 40/44 and for both instruments the cut-off score of 10 will be used to identify alcohol and drug dependency, respectively. Psychometric properties of both instruments are satisfactory [68].

##### Substance use & life events: adverse life-events

Based on Brugha’s list of threatening experiences (LTE) [69], that includes 12 distinct negative life-events, the life-time and past year occurrence of negative life-events will be assessed with *yes/no* items. Items are, for example, *having been severely ill or a victim of violence*, *the death of a parent, sibling or child*, and *having been fired*. Besides the separate items, a sum score will be computed with higher scores indicating higher exposure to negative life-events. The LTE is a valid and reliable measure with high test-retest reliability, good agreement with informant information and low internal consistency [70, 71].

##### Subjective appraisal & recovery: quality of life

The Manchester Short Assessment of Quality of Life (MANSA-16) [72] will be administered to assess Quality of life (QoL). With twelve 7-point Likert-type items and four *yes/no* items, participants can indicate their level of satisfaction with respect to several life-domains, including social contact, psychological wellbeing and housing. The MANSA has been demonstrated to have good internal consistency [73]. Higher scores indicate higher QoL.

##### Subjective appraisal & recovery: symptomatic, somatic and personal recovery

The Integrale Herstel Schaal (IHS) [74] will be used to assess recovery. This 13-item instrument is designed to assess recovery among people with an SMI and is suited for the purpose of Routine Outcome Monitoring (ROM). The IHS is a multidimensional instrument that matches the definition of recovery from the patient-perspective and includes the assessment of symptomatic, somatic, social/societal and personal recovery. To our knowledge, the IHS in itself has not been tested for its psychometric properties as the IHS is an assembly of items from existing ROM-instruments, among which the functional remission scale [75], the Health of the Nations Outcome Scales (HoNOS) [75] and the INSPIRE [76]. To be able to compute IHS total scores, HoNOS items retrieved from patient files will be used in addition to the items included in the interview.

##### Subjective appraisal & recovery: personal recovery

The Dutch national recovery scale (in Dutch “Nationale Herstelschaal”) [77], an adaptation of the Questionnaire about the Process of Recovery (QPR) [78], will be used to assess specifically personal recovery. This instrument measures personal recovery on the basis of 26 Likert-type items ranging from 1 = *strongly disagree* to 5 = *strongly agree.* Examples of items are *I feel good about myself*, *My life has a purpose*, *I can manage to actively engage life* and *I have trust in others*. A total score can be computed with a maximum of 4 missing answers and scores range from 26 to 100. Higher scores indicate higher personal recovery. The NHS has been shown to be a user-friendly, reliable and valid instrument [77].

##### Health: physical health

With respect to physical health, both general perceived health (the extent to which people suffer from physical complaints) and physical limitations in daily functioning will be assessed using two subscales of the RAND SF-36 Health Survey [79]. The subscale general health measures self-perceived health, ranging from 1 = *excellent* to 5 = *bad*. The subscale physical functioning measures the level of limitations in daily functioning using 10-items (e.g., lifting groceries, climbing a few stairs) with score categories 1 = *severely limited* and 3 = *not limited*. The subscale physical functioning has an internal consistency of Cronbach’s α = .92, and good test-retest reliability after 2 (r = .82) and 6 months (r = .72), respectively. The sensitivity to change of the general perceived health subscale is satisfactory and it has good convergent validity with other scales. The psychometric properties are sufficient [80].

##### Health: perceived needs for care

The Camberwell Assessment of Need Short Appraisal Schedule (CANSAS) [81], a short version of the Camberwell Assessment of Need (CAN) [82], is used to assess need for care and support. The CANSAS consists of 25 items that concern care needs and care reception in 25 distinct life-domains (e.g., housing, intimate (partner) relations, psychological wellbeing, finances). For each domain, participants indicate if they have a need for care and the extent to which the need is provided for. Each of the domains can be scored with 0 = *no need*, 1 = *met need for care* and 2 = *unmet need for care*. Additionally, for needs for care that are met, participants can indicate if care/support is provided by formal and/or informal care providers. Psychometric properties of the CANSAS were demonstrated to be satisfactory concerning, amongst others, test-retest reliability and interrater reliability [81, 83].

##### Health: psychiatric symptomatology (symptom severity and psychiatric complaints)

The 24-item version of the Brief Psychiatric Rating Scale (BPRS-E) [84–87] will be administered to assess psychopathology severity (and is used to validate the inclusion of participants in the not-in-care group based on the SMI-criteria). In this version of the BPRS [88] 14 items are administered during the interview and 10 are scored by the interviewer after a finished interview. Each item is scored on an anchored 7-point scale varying from 1 = *not reported/not observed* to 4 = *moderate* and 7 = *very severe*. Scores indicate the presence and severity of psychiatric symptoms observed during the interview or reported by the participant with scores 2–3 indicating non-pathological intensity and scores 4–7 indicating pathological intensity of the symptom at hand. Besides the calculation of a total BPRS-score, the following subscales can be calculated: positive symptoms, negative symptoms, depression and anxiety, and disorganization. The BPRS and its subscales are suited for clinical and epidemiological purposes, sensitive for change [89, 90] and overall good psychometric properties of the BPRS have been demonstrated [91].

#### Administrative data

On the basis of informed consent, administrative data from all participants will be retrieved from their patient and/or client files at either Arkin, GGZ inGeest and/or the service providers where “not-in-care” participants will be recruited. These data concern:

- Assessments of general functioning, routinely assessed by mental health services with the 12-item Health of the Nations Outcome Scale (HoNOS) [75]. The HoNOS was developed specifically for the simple, reliable and valid routine assessment of the psychological and social functioning of people with a psychiatric disorder. The HoNOS proved to be sensitive to change. The 12-item HoNOS consists of the subscales behavior problems, psychological and somatic limitations, symptomatology and social problems. HoNOS scores will be considered only if they were obtained 6 months before or after the date of the interview.
- Type and date of crisis-interventions including mental health crisis intervention by Acute care service of the mental health providers as well as the interventions by the Public Health Service Amsterdam [92]; evictions from (social) housing as registered by the social housing providers and the municipal residence registration; compulsory house cleaning and disinfections executed by the PHS and police contacts as either victim or suspect as registered by the police department.
- health care related patient data from Arkin and GGZ inGeest with respect to psychiatric diagnoses, therapy compliance and contacts with other care providers;
- administrative data about the care/support process from other social support/care providers involved, as self-reported by participants;
- registry data from municipal services (e.g., Amsterdam’s social service (in Dutch: “Werk, Participatie en Inkomen”), the care and nuisance hotline (in Dutch: “Meldpunt zorg en woonoverlast”).

### Primary study outcomes

The study has three primary outcome measures: recovery, societal participation, and quality of life. With respect to recovery, the total score of the National Recovery Scale [77] will be used. Concerning societal participation, two separate indicators will be involved: 1) having formal work/daytime activities (yes/no) and 2) the total score of the Social Exclusion Index for Health Surveys (SEI-HS) [55]. Last, quality of life will be operationalized according to the total score of the MANSA-16 [72].

### Bias

Some types of bias will need to be taken into account when interpreting the results.

We set out to include at least 30% of the population with an SMI living within the selected quarters in the city of Amsterdam, recruited from the two main mental health service providers and several primary care- and social support services. Both the selection of quarters and the recruitment method, can introduce selection bias. Although we only include about 8% of the estimated total SMI-population in Amsterdam, we assume that in spreading the selected quarters over the seven residential city districts and recruiting within the two most populated quarters within those districts, we include a representative sample of the SMI-population in Amsterdam. In addition, it is unlikely that the population in other (smaller) quarters differs systemically from the population in the selected quarters on the background- and outcome measures of this study: social-economical and background characteristics of the general population do not vary widely within quarters (in contrast to variance between quarters). As the SMI-population moves within the same (social) housing pool as the general population, selection bias due to sampling in specific quarters is expected to be limited.

### Statistical methods

All cross-sectional socio-demographic data from both baseline and follow-up measurements will be reported in terms of percentages, means with standard deviations, and quartiles. In addition, all study measures (from the instruments administered and from the registry data) will be analyzed descriptively to provide estimates of the relevant proportions within our study population with respect to these variables as (partial) answers to research questions 2, 4 and 5.

In addition, to provide information on the association between experienced need, and the primary outcome variables (research question 1), data collected at baseline will be analyzed using multivariate regression with recovery, societal participation and quality of life as separate dependent variables and total scores on the CANSAS (i.e. number of unmet needs for care), discrimination scale, stigma scale and victimization as independent (predictor) variables. Any potential mediating effects on the associations between predictors and outcomes variables will be investigated by adding age, sex, psychiatric symptoms (BPRS-E), harmful/dependent alcohol and/or drug use (AUDIT and DUDIT respectively), physical health (RAND SF-36) and city-districts as covariates in these analyses.

To answer research question 2, relationships between the outcome measures and distinct CANSAS items, registry data concerning the care process (i.e. treatment compliance, therapeutic alliance, frequency and timeliness of care consults and self-reported support for social problems) from mental health institutions and support/welfare organizations will mainly be reported in terms of percentages and, for obvious comparisons between groups, using regression models and chi-squared tests. Their associated proportion of variance explained Nagelkerke’s R^2^, Phi for 2 × 2 tables and Cramer’s V for larger than 2 × 2 tables will be reported. Phi and Cramer’s V will be interpreted as indicating no or very weak (> 0), weak (>.05), moderate (>.10), strong (>.15) or very strong (>.25) association between variables [93]. Eta-squared and Cohen’s D will be interpreted as very small (0–.19), small (.20–. 49), moderate (.50–.79), large (.80–1.20), very large (1.20–1.99) or huge (2.0) [94].

Research question 3 will be analyzed using data from the baseline and follow-up measurement by means of generalized linear mixed models with repeated measurements of recovery, quality of life and societal participation as dependent variables. The time between both measures will be included as a within-subjects factor. The same set of predictor variables as mentioned above will be treated as time-dependent independent variables. Additionally, the total score on Brugha’s life-events questionnaire will be included as a potential mediator to assess the effect of having experienced adverse life-events in the time period between baseline and follow-up on the associations between predictor and outcome variables. All variables will be checked for potential outliers. Missing values will be dealt with appropriately. Participants with > 80% missing values on all items will be deleted from the final dataset. For most measures, descriptive analyses will be carried out and the proportion of missing values will be reported for each variable of interest. In subsequent statistical analyses, values missing at (completely) random (i.e. MAR and MCAR) will be deleted pair wise. Values that are not missing at random (NMAR) will be imputed with either the mean, median or using multiple imputation, whichever is most appropriate. Concerning outcome measures that are a sum score of items, instructions from the instrument developers will be followed (e.g., impute missing values with the mean value of the non-missing items and calculate a sum score only if < 10% of items are missing).

## Discussion

The study protocol aims at delivering a comprehensive insight into the needs of community-dwelling adults with an SMI. A huge effort will be made to include a representative sample of adults with an SMI who are in care and those who are not in care. The study represents the client perspective in a larger research program about the process, results and effects of deinstitutionalization. The program additionally consists of research projects that focus on the consequences of deinstitutionalization from other perspectives, namely that of the relatives and friends of people with an SMI, the mental health care providers and social services involved, the neighborhoods and fellow residents and local government. In synthesis, the research program aims at providing key elements based on which ambulatory care and support for community-dwelling adults with a severe mental illness best be provided to optimally promote their social recovery.

## Data Availability

The datasets that will be generated during the proposed study will not be made publicly available, because participant confidentiality cannot be fully guaranteed, but will be made available from the corresponding author upon reasonable request.

## Abbreviations

AUDIT: Alcohol use disorder identification test
BMHC: Basic mental health care
BPRS: Brief psychiatric rating scale
CAN: Camberwell assessment of need
CANSAS: Camberwell assessment of need short appraisal schedule
DUDIT: Drug use disorder identification test
EPA: Ernstige psychiatrische aandoening (Dutch translation of SMI)
FAC: Forensic ambulatory care
FACT: Flexible assertive community treatment
GGZ: Geestelijke gezondheidszorg (Dutch for mental health care)
GHB: Gamma hydroxybutyrate
HoNOS: Health of the nations outcome scales
IRS: Integral recovery scale
ISS: Inventory for social support
ISM: Integral safety monitor
JOT: Jellinek outreaching teams
LZA: Langdurig zorgafhankelijken (In English: long-term care-dependent)
MANSA-16: Manchester short assessment of quality of life
MATE: Measurement in the addiction for triage and evaluation
MDMA: 3,4-methyleendioxymethamfetamine (psychostimulant; party drug)
PHS: Public health service Amsterdam
QoL: Quality of life
ROM: Routine outcome monitoring
SEI-HS: Social exclusion index for health surveys
SMI: Severe mental illness
SNQ: Social network questionnaire

## Footnotes

1 The inquiry into participants’ family situation will also be used to collect contact information of up to three family/members and relatives who may be approached as potential participants of the “relatives and friends-study” within the larger research program deinstitutionalization Amsterdam.

## References

[1] Ministerie van Volksgezondheid Welzijn en Sport. Bestuurlijk akkoord toekomst GGZ 2013-2014. 2012. https://www.eerstekamer.nl/overig/20120620/bestuurlijk_akkoord_toekomst_ggz. Accessed 20 June 2021.

[2] Ministerie van Volksgezondheid Welzijn en Sport. Bestuurlijk akkoord geestelijke gezondheidszorg 2014-2014. 2013. https://www.eerstekamer.org/overig/20130421/bestuurlijk_akkoord_geestelijke_gezondheidszorg. Accessed 20 June 2021.

[3] Wet maatschappelijke ondersteuning 2015. Consulted on May 7th, 2021 on https://wetten.overheid.nl/BWBR0035362/2021-07-01.

[4] Wet maatschappelijke ondersteuning 2018. Consulted on May 7th 2021 on https://wetten.overheid.nl/BWBR0015703/2021-01-01.

[5] Kroon H, Knispel A, Hulsbosch L, De Lange A (2021). Landelijke monitor ambulantisering en hervorming langdurige GGZ 2020 [national monitor deinstitutionalization and reform long-term mental health care 2020].

[6] Van Hoof F, Van Erp N, Boumans J, Muusse C (2014). Trendrapportage GGZ: Persoonlijk en maatschappelijk herstel van mensen met ernstige psychische aandoeningen [Trend report mental health care: personal and societal recovery of people with a severe mental illness].

[7] McGurk SR, Mueser KT, DeRosa TJ, Wolfe R (2009). Work, recovery, and comorbidity in schizophrenia: a randomized controlled trial of cognitive remediation. Schizophr Bull.

[8] Sanches SA, Swildens W, van Busschbach JT, van Weeghel J (2019). Identifying social participation subgroups of individuals with severe mental illnesses: a latent class analysis. Soc Psychiatry Psychiatr Epidemiol.

[9] Koenders JF, de Mooij LD, Dekker JM, Kikkert M (2017). Social inclusion and relationship satisfaction of patients with a severe mental illness. Int J Soc Psychiatry.

[10] Huxley P, Thornicroft G (2003). Social inclusion, social quality and mental illness. Br J Psychiatry.

[11] Lloyd C, King R, Moore L (2010). Subjective and objective indicators of recovery in severe mental illness: a cross-sectional study. Int J Soc Psychiatry..

[12] Buckley PF (2006). Prevalence and consequences of the dual diagnosis of substance abuse and severe mental illness. J Clin Psychiatry.

[13] Scott D, Happell B (2011). The high prevalence of poor physical health and unhealthy lifestyle behaviours in individuals with severe mental illness. Issues Ment Health Nurs.

[14] Phelan M, Stradins L, Morrison S (2001). Physical health of people with severe mental illness: can be improved if primary care and mental health professionals pay attention to it. BMJ.

[15] Thornicroft G, Rose D, Kassam A (2007). Discrimination in health care against people with mental illness. Int Rev Psychiatry.

[16] Webber M, Corker E, Hamilton S, Weeks C, Pinfold V, Rose D (2014). Discrimination against people with severe mental illness and their access to social capital: findings from the viewpoint survey. Epidemiology and Psychiatric Sciences.

[17] Alonso J, Buron A, Rojas-Farreras S, de Graaf R, Haro JM, de Girolamo G (2009). Perceived stigma among individuals with common mental disorders. J Affect Disord.

[18] Sheehan L, Nieweglowski K, Corrigan PW, Gaebel W, Rössler W, Sartorius N (2017). Structures and types of stigma. The stigma of mental illness - end of the story?.

[19] Richter D, Hoffmann H (2019). Social exclusion of people with severe mental illness in Switzerland: results from the Swiss health survey. Epidemiol Psychiatr Sci.

[20] Choe JY, Teplin LA, Abram KM (2008). Perpetration of violence, violent victimization, and severe mental illness: balancing public health concerns. Psychiatr Serv.

[21] de Mooij LD, Kikkert M, Lommerse NM, Peen J, Meijwaard SC, Theunissen J (2015). Victimisation in adults with severe mental illness: prevalence and risk factors. Br J Psychiatry.

[22] Kamperman AM, Henrichs J, Bogaerts S, Lesaffre EM, Wierdsma AI, Ghauharali RR (2014). Criminal victimisation in people with severe mental illness: a multi-site prevalence and incidence survey in the Netherlands. PLoS One.

[23] Khalifeh H, Johnson S, Howard LM, Borschmann R, Osborn D, Dean K (2015). Violent and non-violent crime against adults with severe mental illness. Br J Psychiatry.

[24] Meijwaard SC, Kikkert M, de Mooij LD, Lommerse NM, Peen J, Schoevers RA (2015). Risk of criminal victimisation in outpatients with common mental health disorders. PLoS One.

[25] Castelein S, Timmerman ME, van der Gaag M, Visser E (2021). Clinical, societal and personal recovery in schizophrenia spectrum disorders across time: states and annual transitions. Br J Psychiatry.

[26] Leendertse JCP, Wierdsma AI, van den Berg D, Ruissen AM, Slade M, Castelein S (2021). Personal recovery in people with a psychotic disorder: a systematic review and meta-analysis of associated factors. Front Psychiatry.

[27] Mizuno E, Iwasaki M, Sakai I, Kamizawa N (2015). Experiences of community-dwelling persons recovering from severe mental illness. Arch Psychiatr Nurs.

[28] Slade M, Amering M, Farkas M, Hamilton B, O'Hagan M, Panther G (2014). Uses and abuses of recovery: implementing recovery-oriented practices in mental health systems. World Psychiatry.

[29] Leamy M, Bird V, Le Boutillier C, Williams J, Slade M (2011). Conceptual framework for personal recovery in mental health: systematic review and narrative synthesis. Br J Psychiatry.

[30] Peen J, Huijser J, Tas N, Ploeg M, Carpay M, Tuinebreijer W, et al. Resultaten Vignettenstudie EPA Amsterdam [Results vignettes study severe mental illness in Amsterdam]. Amsterdam: Arkin, GGZ inGeest, Cordaan, GGD Amsterdam, HVO Querido, Leger des Heils; 2016. https://www.clientenbelangamsterdam.nl/actueel/nieuws/resultaten-vignettenstudie-epa-amsterdam on 1 June 2021.

[31] Peen J, Theunissen J, Duurkoop P, Kikkert M, Dekker J (2011). Na de extramuralisering; een retrospectief onderzoek naar omvang en zorggebruik van de groep chronische patiënten in de Amsterdamse GGZ [After deinstitutionalization; a retrospective study into the size and care use of chronic SMI patients in Amsterdam]. Tijdschrift voor Psychiatrie.

[32] Theunissen J, Duurkoop P, Kikkert M, Peen J, Dekker J, Na de extramuralisering. II. (2013). Een crosssectionele studie naar psychisch en sociaal functioneren en kwaliteit van leven van een steekproef van chronische psychiatrische patiënten in de Amsterdamse ggz [after deinstitutionalization. II. A cross-sectional study into psychological and social functioning and quality of life among a sample of chronic SMI patients from Amsterdam]. Tijdschrift voor Psychiatrie.

[33] Van Hoof F, Knispel A, Hulsbosch L, Place C, Muusse C, Van Vugt M (2016). Landelijke monitor ambulantisering en hervorming langdurige GGZ [national monitor deinstitutionalization and reform of long-term mental health care].

[34] Delespaul PH (2013). Consensus over de definitie van mensen met een ernstige psychische aandoening (EPA) en Hun aantal in Nederland [consensus about the definiotion of people with a severe mental illness and their number in the Netherlands]. Tijdschrift voor Psychiatrie..

[35] Kessler RC, Barker PR, Colpe LJ, Epstein JF, Gfroerer JC, Hiripi E (2003). Screening for serious mental illness in the general population. Arch Gen Psychiatry.

[36] Lipari RN, Park-Lee E. Substance Abuse and Mental Health Services Administration (SAMHSA), an institution in the USA. https://store.samhsa.gov/product/Key-Substance-Use-and-Mental-Health-Indicators-in-the-United-States-Resultsfrom-the-2018-National-Survey-on-Drug-Use-and-Health/PEP19-5068. Accessed 20 June 2021.

[37] Peen J, Schoevers RA, Beekman AT, Dekker J (2010). The current status of urban-rural differences in psychiatric disorders. Acta Psychiatr Scand.

[38] Perälä J, Suvisaari J, Saarni SI, Kuoppasalmi K, Isometsä E, Pirkola S (2007). Lifetime prevalence of psychotic and bipolar I disorders in a general population. Arch Gen Psychiatry.

[39] Stuurgroep Vignettenstudie EPA. Herziene verantwoording Vignettenstudie EPA Amsterdam 18–65 jaar [Revised justification of the vignettess study into severe mental illness in Amsterdam]. Amsterdam: Arkin, GGZ inGeest, Cordaan, GGD Amsterdam, HVO Querido, Leger des Heils; 2016. https://www.clientenbelangamsterdam.nl/actueel/nieuws/resultaten-vignettenstudie-epa-amsterdam on 1 June 2021.

[40] Van Hoof F, Knispel A, Hulsbosch L, de Lange A, Michon H, Kroon H (2017). Landelijke Monitor Ambulantisering en Hervorming Langdurige GGZ 2017 [National monitor Deinstitutionalization and reform of long-term mental health care 2017].

[41] Substance Abuse and Mental Health Services Administration (2014). SAMHSA's concept of trauma and guidance for a trauma-informed approach. In: 14–4884 HPNS, editor.

[42] American Psychiatric Association. Diagnostic and statistical manual of mental disorders. 5th ed. Arlington: American Psychiatric Association Publishing; 2013.

[43] Ruggeri M, Leese M, Thornicroft G, Bisoffi G, Tansella M (2000). Definition and prevalence of severe and persistent mental illness. Br J Psychiatry.

[44] Zumstein N, Riese F (2020). Defining severe and persistent mental illness—a pragmatic utility concept analysis. Frontiers in Psychiatry.

[45] Dekker JJ, Theunissen J, Van R, Peen J, Duurkoop P, Kikkert M (2010). Victimization of patients with severe psychiatric disorders: prevalence, risk factors, protective factors and consequences for mental health. A longitudinal study BMC Public Health.

[46] Bellis MA, Ashton K, Hughes K, Ford K, Bishop J, Paranjothy S (2015). Adverse childhood experiences and their impact on health-harming behaviours in the welsh adult population. Public Health Wales.

[47] Wijngaarden B (1987). Social network, social steun, gebeurtenissen vragenlijst [social network, social support and life-events questionnaire].

[48] Duurkoop W, Dekker J, Van den Langenberg S, Van Eck L (1991). Het sociale netwerk van chronisch psychiatrische patienten [the social network of chronic patients with an SMI]. Klinische psychiatrie.

[49] van Dam-Baggen C, Huiskes C, Kraaimaat F (1986). Inventory for measuring social involvement. Inventarisatielijst Sociale Betrokkenheid: ISB.

[50] Van Dam-Baggen R, Kraaimaat F (1992). De Inventarisatielijst Sociale Betrokkenheid (ISB): een zelfbeoordelingslijst om sociale steun te meten [the inventory for social support (ISS): a self-report inventory for the measurement of social support]. Gedragstherapie..

[51] De Jong GJ, Van Tilburg T (2010). The De Jong Gierveld short scales for emotional and social loneliness: tested on data from 7 countries in the UN generations and gender surveys. Eur J Ageing.

[52] De Jong-Gierveld J, Kamphuls F (2016). The development of a Rasch-type loneliness scale. Appl Psychol Meas.

[53] van Tilburg TG, Fokkema T (2021). Stronger feelings of loneliness among Moroccan and Turkish older adults in the Netherlands: in search for an explanation. Eur J Ageing.

[54] Hoff S (2014). Replicatie van het meetinstrument voor sociale uitsluiting [replicating the measurement instrument for social exclusion].

[55] Hoff S, Vrooman C (2011). Dimensies van sociale uitsluiting [dimensions of social exclusion].

[56] Vrooman JC, Hoff SJ (2013). The disadvantaged among the Dutch: a survey approach to the multidimensional measurement of social exclusion. Soc Indic Res.

[57] van Bergen AP, Hoff SJ, Schreurs H, van Loon A, van Hemert AM (2017). Social exclusion index-for health surveys (SEI-HS): a prospective nationwide study to extend and validate a multidimensional social exclusion questionnaire. BMC Public Health.

[58] Centraal Bureau voor de Statistiek (2019). Veiligheidsmonitor 2019.

[59] Biderman AD, Reiss AJ (2016). On exploring the "dark figure" of crime. The ANNALS of the American Academy of Political and Social Science.

[60] Kessler RC, Mickelson KD, Williams DR (1999). The prevalence, distribution, and mental health correlates of perceived discrimination in the United States. J Health Soc Behav.

[61] Williams DR, Mohammed SA (2009). Discrimination and racial disparities in health: evidence and needed research. J Behav Med.

[62] King M, Dinos S, Shaw J, Watson R, Stevens S, Passetti F (2007). The stigma scale: development of a standardised measure of the stigma of mental illness. Br J Psychiatry.

[63] Brohan E, Slade M, Clement S, Thornicroft G (2010). Experiences of mental illness stigma, prejudice and discrimination: a review of measures. BMC Health Serv Res.

[64] Schippers GM, Broekman TG, Buchholz A, Koeter MW, Van Den Brink W (2010). Measurements in the addictions for triage and evaluation (MATE): an instrument based on the World Health Organization family of international classifications. Addiction..

[65] American Psychiatric Association (2000). DSM-IV-TR: diagnostic and statistical manual of mental disorders-IV-text revision.

[66] Babor TF, Higgins-Biddle JC, Saunders JB, Monteiro MG (2001). The alcohol use disorders identification test (AUDIT): guidelines for use in primary care.

[67] Berman AH, Bergman H, Palmstierna T, Schlyter F (2005). Evaluation of the drug use disorders identification test (DUDIT) in criminal justice and detoxification settings and in a Swedish population sample. Eur Addict Res.

[68] Hildebrand M (2015). The psychometric properties of the drug use disorders identification test (DUDIT): a review of recent research. J Subst Abus Treat.

[69] Brugha T, Bebbington P, Tennant C, Hurry J (1985). The list of threatening experiences: a subset of 12 life event categories with considerable long-term contextual threat. Psychol Med.

[70] Brugha TS, Cragg D (1990). The list of threatening experiences: the reliability and validity of a brief life events questionnaire. Acta Psychiatr Scand.

[71] Motrico E, Moreno-Küstner B, de Dios LJ, Torres-González F, King M, Nazareth I (2013). Psychometric properties of the list of threatening experiences—LTE and its association with psychosocial factors and mental disorders according to different scoring methods. J Affect Disord.

[72] Priebe S, Huxley P, Knight S, Evans S (1999). Application and results of the Manchester short assessment of quality of life (MANSA). Int J Soc Psychiatry..

[73] Bjorkman T, Svensson B (2005). Quality of life in people with severe mental illness. Reliability and validity of the Manchester short assessment of quality of life (MANSA). Nordic Journal of Psychiatry.

[74] Swildens W, Nugter A, Schaefer B, Visser E. De Integrale Herstel Schaal (IHS): validering van een nieuw instrument voor routine outcome monitoring bij mensen met Ernstige Psychische Aandoeningen [the integral recovery scale: validation of a new instrument for routine outcome monitoring among people with a severe mental illness]. Amsterdam: Hogeschool InHolland; 2017.

[75] Wing J, Beevor A, Curtis R, Park S, Hadden J, Burns A (1998). Health of the nation outcome scales (HoNOS): Research and Development. Br J Psychiatry.

[76] Williams J, Leamy M, Bird V, Le Boutillier C, Norton S, Pesola F (2015). Development and evaluation of the INSPIRE measure of staff support for personal recovery. Soc Psychiatry Psychiatr Epidemiol.

[77] van Gestel JAWM, van Veldhuijsen RESR, van Weeghel Jaap, van Nieuwenhuizen Chijs. Verslag psychometrische evaluatie van de Nationale Herstelschaal [Report on the psychomteric evaluation of the National Recovery Scale]. Tranzo, Scientific center for care and wellbeing. Tilburg: Tilburg University/SBWU. 2015.

[78] Neil ST, Kilbride M, Pitt L, Nothard S, Welford M, Sellwood W (2009). The questionnaire about the process of recovery (QPR): a measurement tool developed in collaboration with service users. Psychosis.

[79] Ware JE (2000). SF-36 health survey update. Spine (Phila Pa 1976).

[80] Van der Zee K, Sanderman R. Het meten van de algemene gezondheidstoestand met de RAND-36, een handleiding. [Measuring general health with the RAND-36: a manual]. Groningen: Northern Centre for Health Care Research, University of Groningen, the Netherlands; 1993.

[81] Trauer T, Tobias G, Slade M (2008). Development and evaluation of a patient-rated version of the Camberwell assessment of need short appraisal schedule (CANSAS-P). Community Ment Health J.

[82] Phelan M, Slade M, Thornicroft G, Dunn G, Holloway F, Wykes T (1995). The Camberwell assessment of need: the validity and reliability of an instrument to assess the needs of people with severe mental illness. Br J Psychiatry.

[83] Slade M, Beck A, Bindman J, Thornicroft G, Wright S (1999). Routine clinical outcome measures for patients with severe mental illness: CANSAS and HoNOS. Br J Psychiatry.

[84] Burger GK, Calsyn RJ, Morse GA, Klinkenberg WD, Trusty ML (1997). Factor structure of the expanded brief psychiatric rating scale. J Clin Psychol.

[85] Dingemans PM, Linszen DH, Lenior ME, Smeets RM (1995). Component structure of the expanded brief psychiatric rating scale (BPRS-E). Psychopharmacology.

[86] Lukoff D, Nuechterlein K, Ventura J (1986). Manual for the expanded brief psychiatric rating scale. Schizophr Bull.

[87] Overall JE, Gorham DR (2016). The brief psychiatric rating scale. Psychol Rep.

[88] Leucht S, Kane JM, Kissling W, Hamann J, Etschel E, Engel R (2005). Clinical implications of brief psychiatric rating scale scores. Br J Psychiatry.

[89] Ballerini A, Boccalon R, Boncompagni G, Casacchia M, Margari F, Minervini L (2007). An observational study in psychiatric acute patients admitted to general hospital psychiatric wards in Italy. Ann General Psychiatry.

[90] Zanello A, Berthoud L, Ventura J, Merlo MC (2013). The brief psychiatric rating scale (version 4.0) factorial structure and its sensitivity in the treatment of outpatients with unipolar depression. Psychiatry Res.

[91] Ruggeri M, Koeter M, Schene A, Bonetto C, Vàzquez-Barquero JL, Becker T (2005). Factor solution of the BPRS-expanded version in schizophrenic outpatients living in five European countries. Schizophr Res.

[92] Steele CM, Josephs RA (1990). Alcohol myopia: its prized and dangerous effects. Am Psychol.

[93] Akoglu H (2018). User's guide to correlation coefficients. Turk J Emerg Med.

[94] Cohen J (2013). Statistical power analysis for the behavioral sciences.

